# Dual optimization in single-isocenter radiosurgery for multiple brain metastases: a dosimetric planning study

**DOI:** 10.3389/fonc.2026.1901196

**Published:** 2026-08-05

**Authors:** Alparslan Serarslan, Rana Elif Yıldız, Telat Aksu, Nilgün Özbek Okumuş, Deniz Meydan, Bilge Gürsel

**Affiliations:** Department of Radiation Oncology, Faculty of Medicine, Ondokuz Mayıs University, Samsun, Türkiye

**Keywords:** brain metastases, computer-assisted radiotherapy planning, linac radiosurgery, radiation dosimetry, radiotherapy, stereotactic radiation therapy, stereotactic radiosurgery, volumetric modulated arc therapy

## Abstract

**Background:**

This study evaluated a dual-optimization strategy for single-isocenter multiple-target stereotactic radiosurgery (SIMT-SRS) in patients with multiple brain metastases, combining target-anchored isocenter placement with individualized dose prescription for each planning target volume (PTV).

**Methods:**

Three volumetric modulated arc therapy-based SIMT-SRS planning techniques were compared in 22 patients harboring 5–10 brain metastases: geometric center isocenter with composite dose prescription (GCI-Comp), target-anchored isocenter with composite dose prescription (TAI-Comp), and target-anchored isocenter with individual dose prescription (TAI-Ind). Dosimetric parameters for PTVs and organs at risk (OARs) were extracted and compared using the Friedman test followed by Bonferroni-corrected *post hoc* analyses.

**Results:**

All three techniques achieved equivalent target coverage (V95% > 99%, *p* = 0.202), plan quality (Q_RTOG_, *p* = 0.385), and conformity (CI_Paddick_, *p* = 0.546). Compared to GCI-Comp and TAI-Comp, TAI-Ind produced significantly higher intratumoral dose escalation (PTV–D_max_: 2025.60 vs. 2113.80 vs. 2241.34 cGy, *p* < 0.001) while achieving a significantly steeper dose gradient (R_50%_: 13.43 vs. 12.07 vs. 10.31; GI_Paddick_: 12.91 vs. 12.10 vs. 9.41; both *p* < 0.001). TAI-Ind also resulted in the lowest normal brain dose exposure (V12Gy: 35.53 cm^3^; D_50%_: 322.36 cGy) and significantly reduced maximum doses to the hippocampus, brainstem, spinal canal, and bilateral cochleae (all *p* < 0.001). These dosimetric advantages were achieved at the expense of requiring a higher number of monitor units (6013.84 vs. 6494.89 vs. 7268.92, *p* < 0.001).

**Conclusions:**

The TAI-Ind dual-optimization strategy provided greater intratumoral dose escalation and more favorable OARs sparing compared with the composite-prescription techniques, without compromising target coverage. This approach represents a promising strategy for SIMT-SRS planning in patients with multiple brain metastases, warranting further clinical validation.

## Introduction

Brain metastases (BMs) are the most common intracranial malignancies in adults and represent a critical determinant of patient prognosis and quality of life. Their management poses a complex oncological challenge that requires a multidisciplinary approach (1). Effective treatment of BMs relies on the integration of neurosurgical resection, brain-penetrant systemic therapies, and various radiotherapy (RT) modalities (2). RT techniques for BMs have evolved from whole-brain RT to the highly focal, neurocognitive-sparing precision of stereotactic radiosurgery (SRS) (3).

SRS, defined as the highly conformal delivery of ablative ionizing radiation to precisely delineated intracranial targets, has evolved beyond its historical application to a limited number of lesions and has become a key therapeutic option even for patients with extensive multiple BMs, provided that the cumulative tumor volume remains acceptable. By maximizing local tumor control while minimizing neurocognitive decline (3), SRS offers a highly effective treatment approach that can be delivered using several different technological platforms (4). Although the fundamental dosimetric and radiobiological principles of SRS are consistently applied across dedicated treatment systems with comparable clinical efficacy, modern linear accelerators (LINACs) have increasingly become central to neuro-oncological practice because of their versatility, widespread availability, and efficient treatment delivery (5, 6). LINAC-based SRS has transformed the management of multiple BMs by overcoming the prolonged treatment times and sequential setup requirements associated with conventional multi-isocenter approaches (7, 8). Through advanced volumetric modulation techniques, LINACs enable the simultaneous treatment of multiple lesions using single-isocenter multiple-target SRS (SIMT-SRS) (3, 9).

While positioning a single isocenter at the geometric center of multiple metastases substantially improves treatment efficiency and patient comfort, this strategy inherently increases susceptibility to off-axis rotational errors and the island-blocking effect. These limitations may lead to increased low-dose spillage into healthy brain tissue and inadequate peripheral target coverage (10). Consequently, achieving steep dose gradients while minimizing radiation exposure to organs at risk (OARs) remains a significant challenge in SIMT-SRS.

This dosimetric study aimed to evaluate a refined planning strategy in which the isocenter is shifted from the conventional geometric center to the metastasis located nearest to it, while simultaneously implementing individualized dose prescriptions for each planning target volume (PTV) rather than prescribing dose based on the total target volume. We hypothesized that this dual-optimization approach would enhance dose fall-off and improve OARs sparing without compromising target coverage.

## Methods

### Ethics statement

This study was conducted in accordance with the Declaration of Helsinki and was approved by the local ethics committee of the Faculty of Medicine, Ondokuz Mayıs University, Samsun, Türkiye (application number: 2025000338; approval date: June 11, 2025; approval number: 2025/338). Written informed consent was obtained from all patients prior to participation in the study.

### Patients

In total, 22 patients with histopathologically confirmed primary cancer and BMs were enrolled in this study. The inclusion criteria were: the presence of 5–10 BMs (11), a maximum diameter of ≤ 2 cm for the largest metastatic lesion (11), and a cumulative PTV of all metastatic lesions of 4–20 cm^3^ per patient (12), considering the volumetric and technical constraints of LINAC-based SRS. To ensure cohort homogeneity, patients were excluded if they did not meet the inclusion criteria; had any metastatic lesion located within 5 mm of the brainstem, hippocampus, or optic pathways; or had a history of previous neurosurgical intervention for BMs, irrespective of the clinical indication. The original SRS treatments of the included patients were delivered between September 2018 and June 2023. For the purposes of this dosimetric replanning study, all technique-specific plans were generated between July and August 2025, using each patient’s original simulation imaging, target volumes, and OARs contours.

The sample size was determined based on a power analysis using data reported by Chea et al. (13). The power of their study was calculated to be 99% based on the gradient index (GI) values [Brainlab Elements Multiple Brain Mets: 4.09 ± 1.14; Gamma Knife: 3.22 ± 0.55; pairwise comparison, *p* < 0.001], an alpha level of 0.05, and a sample size of 20 patients. To strengthen the statistical robustness of our analysis further, we included 22 patients in the present study.

### Simulation

All patients were immobilized in the supine position using a non-invasive thermoplastic head mask (IMRT Head and Neck Mask, Biomalzeme Limited Company, Türkiye). RT planning was performed using a three-dimensional computed tomography simulator (Aquilion LB; Toshiba Medical Systems, Otawara, Japan). Contrast-enhanced simulation scans were acquired with a high-resolution slice thickness of 1 mm, providing an optimal foundation for subsequent multimodal image co-registration. Following image acquisition, the datasets were transferred to the treatment planning system (Eclipse version 13.7.16; Varian Medical Systems) via a Digital Imaging and Communications in Medicine network.

### Image co-registration and target delineation

High-resolution stereotactic magnetic resonance imaging was performed using a 1.5-T scanner, with acquisition of 1-mm-thick volumetric T1-weighted sequences following administration of a double dose of gadolinium contrast (T1W + C). The contrast-enhanced magnetic resonance imaging datasets were subsequently co-registered with the planning computed tomography images. Following accurate image fusion, the clinical target volume was defined as equivalent to the gross tumor volume (GTV), given the limited microscopic infiltration typically associated with BMs. To account for setup and geometric uncertainties, the PTV was generated by applying a uniform isotropic 2-mm expansion to the GTV (14, 15).

### OARs delineation

OARs were delineated according to the consensus-based guidelines of the European Particle Therapy Network (16). The normal brain was defined as the whole-brain volume excluding the GTV. The optic pathway was contoured as a single structure encompassing the optic nerves and optic chiasm. The brainstem included the midbrain and pons, whereas the medulla oblongata was assigned to the spinal canal. In addition, the hippocampus, cochleae, lenses, and orbits were contoured as separate structures (16–18).

### RT planning

All plans were generated by the same radiation oncologist and medical physicist using a standardized protocol to ensure methodological consistency across patients and techniques. For the SIMT-SRS of multiple BMs, volumetric modulated arc therapy (VMAT) was used as the primary treatment delivery technique. All treatment plans were generated using the Eclipse treatment planning system (version 17.0; Varian Medical Systems, Palo Alto, CA, USA). Treatments were planned with 6-MV flattening filter-free photon beams at a maximum dose rate of 1400 monitor units (MU) per minute using a Varian Millennium 120 multileaf collimator (MLC), which features leaf widths of 5 mm in the central region and 10 mm in the peripheral region. To evaluate the impacts of isocenter placement and dose prescription strategies, three distinct planning techniques were developed and compared.

**Figure 1:**
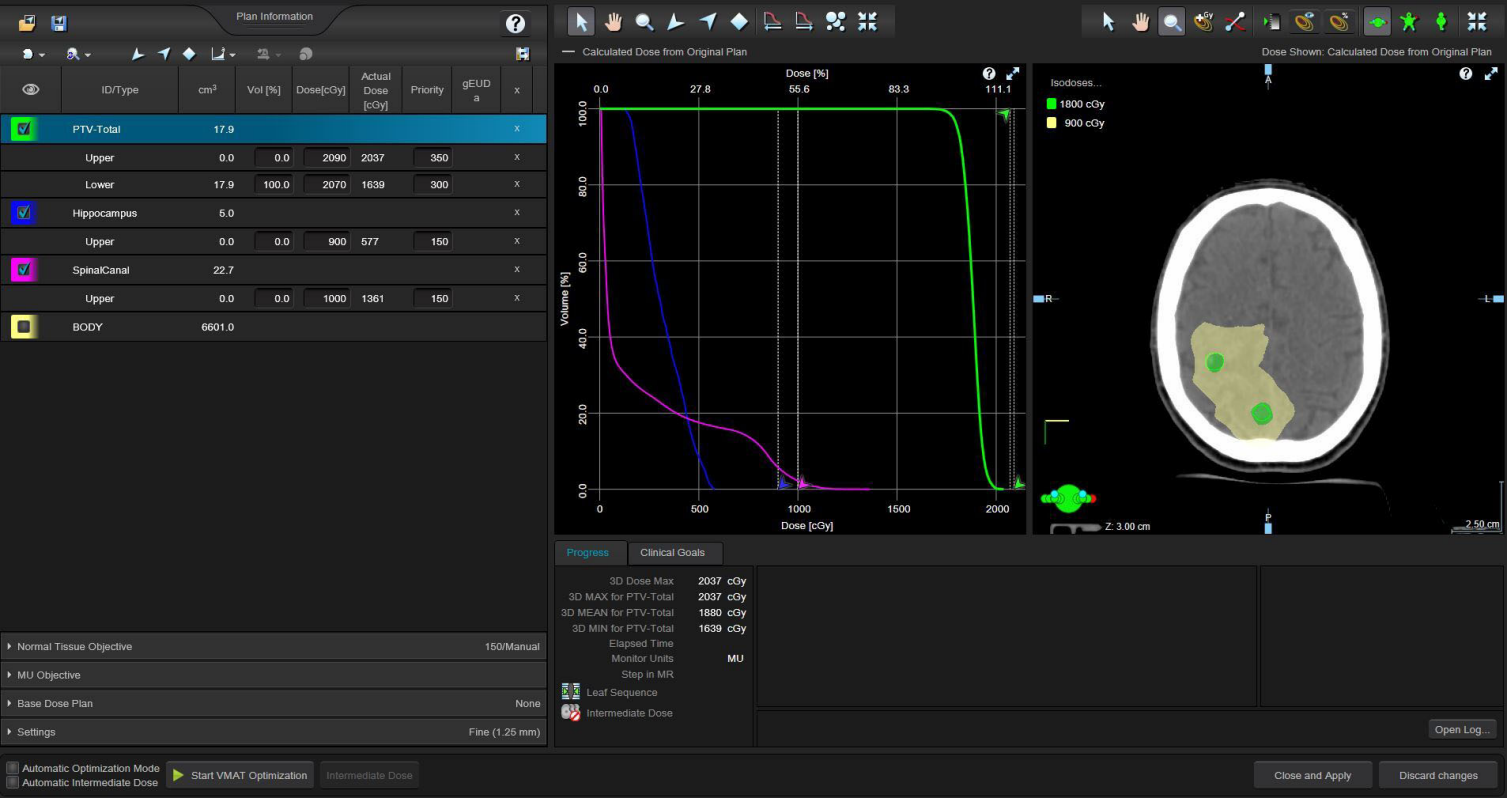
The optimization phase of geometric center isocenter with composite dose prescription (GCI-Comp) technique for a patient. In the planning system, the calculated doses are shown in green for total PTV (range, 1639–2037 cGy), blue for hippocampus-D_max_ (577 cGy), magenta for spinal canal D_max_ (1361 cGy) and dose bridging between metastases is also seen in the axial section.

Geometric Center Isocenter with Composite Dose Prescription (GCI-Comp): In this baseline approach, a single isocenter was automatically assigned by the treatment planning system to the geometric center of all targets. During optimization, the prescribed dose was defined globally to the composite PTV (total PTV) (**Figure 1**).

Target-Anchored Isocenter with Composite Dose Prescription (TAI-Comp): In this technique, the isocenter was automatically positioned at the center of the metastasis located closest to the overall geometric center. Similar to the GCI-Comp approach, the prescribed dose was defined to the composite PTV (total PTV) during optimization.

**Figure 2:**
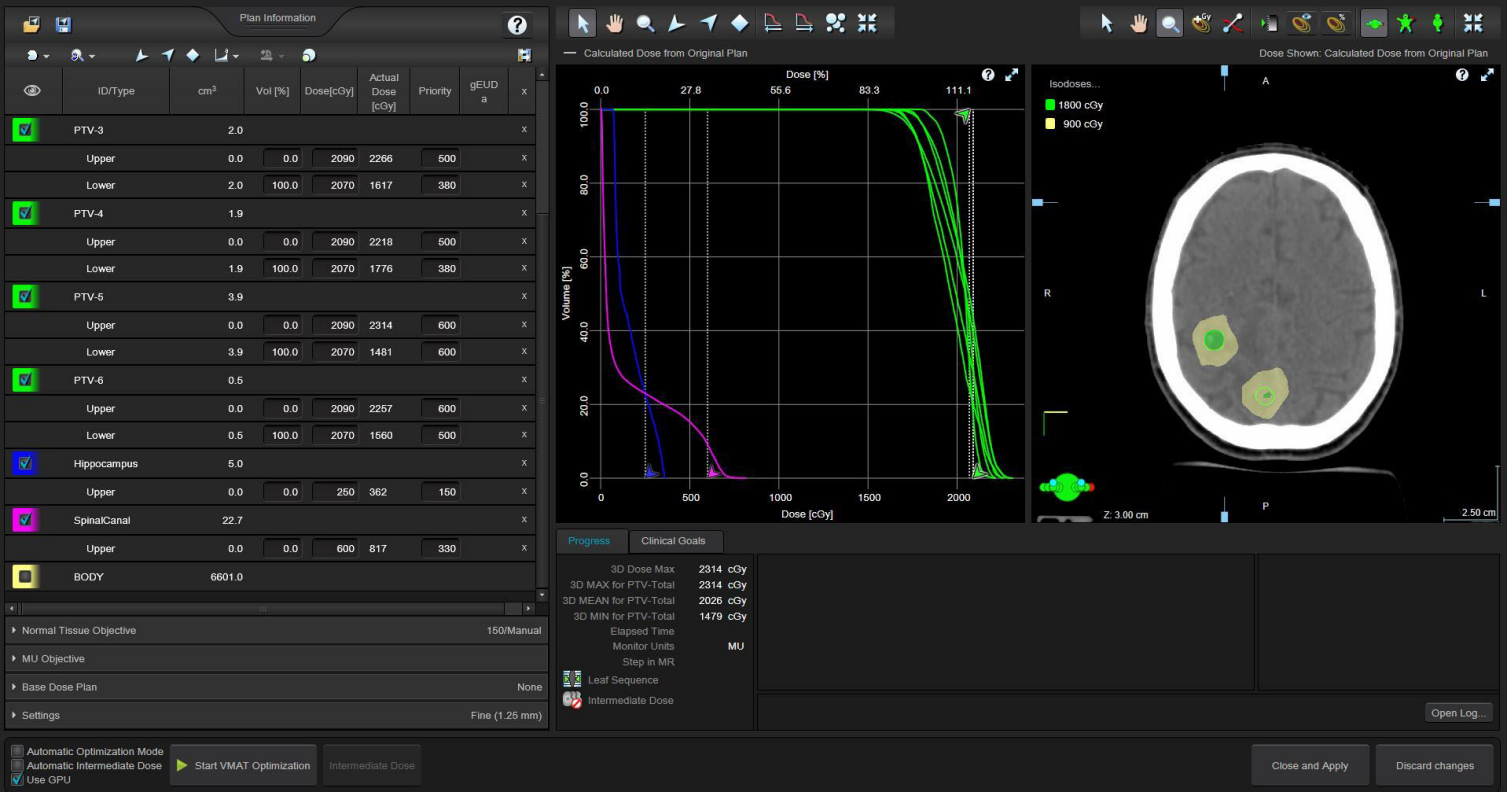
The optimization phase of target-anchored ısocenter with ındividual dose prescription (TAI-Ind) technique for the same patient in **Figure 1**. In the planning system, the calculated doses are shown in green for individual PTVs, or from PTV-1 to PTV-5, (range, 1479–2314 cGy), blue for hippocampus-D_max_ (362 cGy), magenta for spinal canal D_max_ (817 cGy) and in the axial section, it is seen that there is no dose bridging between metastases.

Target-Anchored Isocenter with Individual Dose Prescription (TAI-Ind): In this refined approach, the isocenter was likewise automatically assigned to the metastasis nearest to the geometric center. However, unlike the GCI-Comp and TAI-Comp techniques, optimization priorities were assigned such that the prescribed dose was defined separately for the individual PTV of each metastasis rather than for a single composite target volume (total PTV) (**Figure 2**).

To ensure optimal target coverage and delivery efficiency across all techniques, VMAT plans were configured using three coplanar full arcs. The first arc rotated clockwise from 181° to 179° with a collimator angle of 15°, followed by a counterclockwise arc from 179° to 181° with a collimator angle of 345°. The third arc rotated clockwise from 181° to 179° with a collimator angle of 90°. Importantly, the 90°collimator angle was manually selected to mitigate the island-blocking phenomenon and minimize inter-target dose bridging.

Plan optimization was performed using the Photon Optimizer algorithm (version 17.0; Varian Medical Systems, Palo Alto, CA, USA), which dynamically modulates MLC leaf positions, dose rates, and gantry speeds. To achieve a steep dose gradient and maximize sparing of the surrounding healthy brain tissue, the Normal Tissue Objective tool was applied during optimization. In accordance with the clinical recommendations of Hartgerink et al. (12), a single-fraction prescription dose of 18 Gy was prescribed for all plans, as the total PTV of the metastatic lesions was 4–20 cm^3^. Dose prescription was normalized to ensure that at least 95% of the PTV received the prescribed dose. In addition, plans were prescribed to the 95% isodose line to facilitate dose escalation within the central tumor volume. Final volumetric dose calculations were performed using the anisotropic analytical algorithm with a high-resolution calculation grid size of 1 mm, and heterogeneity corrections were applied to all dose calculations. PTV D_2%_ values were permitted to reach up to 140% of the prescribed dose.

To quantify the spatial relationship between the isocenter and each irradiated metastasis, the Euclidean distance was calculated for both isocenter placement strategies. For a given metastasis *i* with centroid coordinates (*x*_i_, *y*_i_, *z*_i_) and an isocenter located at (*x*_0_, *y*_0_, *z*_0_), the distance *d*_i_ was defined as: *d*_i_ = |s^→^_i_ − s^→^_0_| = √[(*x*_i_ − *x*_0_)² + (*y*_i_ − *y*_0_)² + (*z*_i_ − *z*_0_)²] where *d*_i_ is the Euclidean distance between the isocenter and metastasis *i*; s^→^_0_ is the position vector of the isocenter, with coordinates (*x*_0_, *y*_0_, *z*_0_); and s^→^_i_ is the position vector of the centroid of metastasis *i*, with coordinates (*x*_i_, *y*_i_, *z*_i_) (19).

### Dosimetric evaluation and plan comparison

To assess target coverage and dose distribution quality systematically, a comprehensive set of dosimetric parameters was extracted from the dose–volume histograms of each generated plan. For the PTV, the percentage of the volume receiving at least 95% of the prescribed dose (PTV–V_95%_) was initially evaluated. This was followed by the assessment of the near-minimum dose (PTV–D_98%_), absolute maximum dose (PTV–D_max_), near-maximum dose (PTV–D_2%_), and median dose (PTV–D_50%_). In addition, the mean dose (PTV–D_mean_), absolute minimum dose (PTV–D_min_), and the minimum doses covering 99% and 100% of the PTV (PTV–D_99%_ and PTV–D_100%_, respectively) were recorded.

To quantify intermediate- and low-dose spillage, the R_50%_ metric, defined as the ratio of the 50% prescription isodose volume (PIV) to the PTV, was calculated. Lower R_50%_ values indicate a steeper dose gradient and more effective sparing of the surrounding healthy brain parenchyma, whereas higher values reflect greater intermediate-dose spillage beyond the target boundaries (20). Plans’ quality of coverage was subsequently assessed using the Radiation Therapy Oncology Group quality of coverage index (Q_RTOG_), calculated as Q_RTOG_ = I_min_/RI, where I_min_ represents the minimum dose within the target volume and RI denotes the prescription dose. A Q_RTOG_ value ≥ 0.9 was considered protocol compliant, values between 0.8 and 0.9 represented a minor deviation, and values < 0.8 were classified as major deviations from acceptable planning standards.

To account for the spatial overlap between target and prescription isodose volumes more accurately, the Paddick Conformity Index (CI_Paddick_) was calculated using the formula CI_Paddick_ = (TVPIV)^2^/(TV × PIV), where TVPIV denotes the target volume covered by the prescription isodose. An ideal CI_Paddick_ value is 1, with lower values indicating reduced conformity (21). The steepness of dose fall-off outside the target was quantified using the Paddick GI (GI_Paddick_), defined as the ratio of the volume receiving 50% of the prescribed dose (PIV_half_) to the prescription isodose volume, expressed as GI_Paddick_ = PIV_half_/PIV. Lower GI_Paddick_ values indicate a steeper dose gradient. Although a value of approximately 3.0 is considered optimal for single-target radiosurgery, higher values are generally expected in SIMT-SRS because of the geometric complexity associated with inter-target dose bridging (22–24). Treatment delivery efficiency and plan complexity were ultimately represented by the total number of MU. The dose constraints applied to OARs are presented in **Table 1**. Because target coverage was prioritized in this dosimetric study, no compromises in PTV coverage were accepted, even when OARs dose constraints were exceeded.

**Table 1:** Dose constraints for organs at risk.

Organ	Constraint	Optimal	Mandatory
Normal brain^¶^	D10 cm^3^	< 1200 cGy	-
	D_50%_	< 500 cGy	-
Hippocampus	D_max_	-	< 665 cGy
	D_100%_	-	< 421 cGy
Optic pathway	D_max_	–	< 800 cGy
Cochlea	D_mean_	< 400 cGy	< 900 cGy
	D_max_	-	< 900 cGy
Lens	D_max_	< 150 cGy	–
Orbit	D_max_	< 800 cGy	–
Brainstem†	D_max_	< 1000 cGy	< 1500 cGy
Spinal canal‡	D_max_	< 1000 cGy	< 1400 cGy

D10 cm^3^ is the minimum dose to the specified volume (D10 cm^3^) of the organ that receive the highest dose; D_50%_, dose received by 50% of the structure volume; D_100%,_ dose received by 100% of the structure volume; D_max_, minimum dose to the 0.1 cm^3^ volume of the organ receiving the highest doses; ^¶^normal brain is whole brain minus gross tumor volume; ^†^the brainstem consists of the midbrain and pons but without medulla oblongata; ^‡^spinal canal also includes the medulla oblongata (17, 18).

### Statistical analysis

Descriptive data were summarized as the mean ± standard deviation (minimum–maximum). Dosimetric differences among the three treatment techniques were evaluated using the Friedman test, a non-parametric statistical method for repeated measures. When the overall test indicated statistical significance (*p* < 0.05), *post hoc* pairwise comparisons were performed using the Wilcoxon signed-rank test. To account for multiple comparisons and reduce the risk of type I error, Bonferroni correction was applied, resulting in an adjusted significance threshold of α = 0.0167. All statistical analyses were performed using R software, version 4.5.2 (R Foundation for Statistical Computing, Vienna, Austria).

## Results

### Characteristics of metastases

In this dosimetric study, three distinct SRS planning techniques were evaluated and compared. The analysis included 22 patients with a total of 154 intact BMs (median = 7; range, 5–10). The morphological and volumetric characteristics of the treated lesions are presented in **Table 2**. The Euclidean distance between the isocenter and each treated metastasis (n = 154) was 5.74 ± 1.14 cm (range, 1.29 – 8.59 cm) for the geometric center isocenter and 6.33 ± 3.66 cm (range, 0.00 – 15.17 cm) for the target-anchored isocenter (p = 0.016), the latter reflecting a distance of 0 cm for the anchor lesion itself.

**Table 2:** Characteristics of the metastases.

Characteristic	Minimum	Maximum	Mean ± SD
Major axis diameter (cm, n = 22)	0.70	2.00	1.56 ± 0.35
GTV per metastasis (cm^3^, n = 154)	0.01	3.30	0.50 ± 0.60
GTV_Total_ per patient (cm^3^, n = 22)	1.48	8.00	3.54 ± 1.71
PTV per metastasis (cm^3^, n = 154)	0.20	6.40	1.40 ± 1.31
PTV_Total_ per patient (cm^3^, n = 22)	5.40	18.2	9.84 ± 3.49

SD, standard deviation; cm^3^, cubic centimeter; cm, centimeter; GTV, gross tumor volume; PTV, planning target volume.

### Dosimetric outcomes of PTVs

The comparative dosimetric outcomes for the PTVs across the three planning techniques are presented in **Table 3**. All three techniques—GCI-Comp, TAI-Comp, and TAI-Ind—provided excellent and statistically comparable target coverage. This was demonstrated by PTV–V_95%_ values consistently exceeding 99%, with no significant differences among the techniques (*p* = 0.202). In accordance with established dose-reporting guidelines, this finding was further supported by comparable absolute minimum target doses (PTV–D_min_, *p* = 0.385; PTV–D_100%_, *p* = 0.610) and near-minimum target doses (PTV–D_99%_, *p* = 0.113). Although PTV–D_98%_ demonstrated a statistically significant stepwise reduction across the three techniques (*p* = 0.002), values remained consistently high. Moreover, overall plan quality and conformality were maintained, with no significant differences observed in Q_RTOG_ (*p* = 0.385) or CI_Paddick_ (*p* = 0.546).

**Table 3:** Dosimetric parameters for planning target volumes in each technique.

	Mean ± SD (minimum – maximum)			Global P
	GCI-Comp	TAI-Comp	TAI-Ind	
PTV-V_95%_ (%)	99.50 ± 0.25 (99.10-99.92)	99.52 ± 0.22 (98.98-99.93)	99.37 ± 0.37 (98.65-99.90)	0.202
PTV-D_98%_ (cGy)	1768.26 ± 7.29^a^ (1756.00-1780.00)	1762.73 ± 5.99^b^ (1750.00-1773.00)	1755.56 ± 10.47^c^ (1737.00-1769.30)	0.002
PTV-D_max_ (cGy)	2025.60 ± 22.80^a^ (1979.40-2071.00)	2113.80 ± 34.99^b^ (2026.00-2188.00)	2241.34 ± 129.78^c^ (2049.50-2478.20)	<0.001
PTV-D_2%_ (cGy)	1967.29 ± 15.12^a^ (1941.60-1994.00)	2044.44 ± 31.32^b^ (1960.00-2090.00)	2160.90 ± 120.61^c^ (1926.26-2369.00)	<0.001
PTV-D_50%_ (cGy)	1894.85 ± 11.57^a^ (1878.00-1913.00)	1936.57 ± 17.71^b^ (1894.00-1962.00)	2011.66 ± 78.70^c^ (1906.60-2247.60)	<0.001
PTV-D_mean_ (cGy)	1887.11 ± 9.54^a^ (1875.00-1903.00)	1926.58 ± 16.09^b^ (1886.00-1950.00)	1986.86 ± 56.81^c^ (1899.00-2089.70)	<0.001
PTV-D_min_ (cGy)	1550.46 ± 54.39 (1442.70-1655.00)	1564.94 ± 47.33 (1467.30-1640.50)	1556.74 ± 56.18 (1459.00-1654.30)	0.385
PTV-D_99%_ (cGy)	1739.05 ± 14.76 (1711.00-1767.00)	1734.91 ± 10.47 (1707.00-1748.10)	1726.89 ± 19.55 (1687.00-1760.00)	0.113
PTV-D_100%_ (cGy)	1570.13 ± 47.76 (1445.59-1655.45)	1570.51 ± 47.26 (1469.80-1640.00)	1565.46 ± 63.23 (1456.00-1699.00)	0.610
R_50%_	13.43 ± 4.74^a^ (7.88-28.31)	12.07 ± 3.03^a^ (8.63-20.65)	10.31 ± 3.85^b^ (5.77-22.69)	<0.001
Q_RTOG_	0.86 ± 0.03 (0.80-0.92)	0.87 ± 0.03 (0.82-0.91)	0.86 ± 0.03 (0.81-0.92)	0.385
CI_Paddick_	0.79 ± 0.05 (0.67-0.88)	0.78 ± 0.05 (0.66-0.86)	0.78 ± 0.07 (0.55-0.86)	0.546
GI_Paddick_	12.91 ± 4.04^a^ (8.03-23.52)	12.10 ± 3.12^a^ (7.39-20.65)	9.41 ± 2.58^b^ (5.58-15.71)	<0.001
Monitor unit	6013.84 ± 615.61^a^ (5289.00-7560.00)	6494.89 ± 752.16^b^ (5540.60-8736.00)	7268.92 ± 1076.94^c^ (5135.3-9622.0)	<0.001

SD, standard deviation; GCI-Comp, Geometric Center Isocenter with Composite Dose Prescription; TAI-Comp, Target-Anchored Isocenter with Composite Dose Prescription; TAI-Ind, Target-Anchored Isocenter with Individual Dose Prescription; Q_RTOG_, RTOG Quality of Coverage; GI_Paddick_, Paddick Gradient Index; a, b, or c, in the comparison between two groups, if the groups have the same letter, there is no statistically significant difference, but if the same letter is not found, there is a statistically significant difference.

Building upon the TAI-Comp approach, which strategically positions the isocenter at the metastasis nearest to the geometric center, the incorporation of individual PTV prioritization in the TAI-Ind technique represented a successful dual-optimization strategy. This approach had a pronounced effect on intratumoral dose heterogeneity and dose-gradient steepness. Specifically, TAI-Ind enabled deliberate dose escalation within the PTVs, as reflected by significantly higher maximum (PTV–D_max_, *p* < 0.001), near-maximum (PTV–D_2%_, *p* < 0.001), median (PTV–D_50%_, *p* < 0.001), and mean doses (PTV–D_mean_, *p* < 0.001), all of which demonstrated a statistically significant stepwise increase across the evaluated techniques.

Importantly, this enhanced central dose deposition did not compromise normal tissue sparing. On the contrary, it resulted in a substantially steeper dose fall-off, as evidenced by significantly lower R_50%_ (10.31 ± 3.85) and GI_Paddick_ (9.41 ± 2.58) values in the TAI-Ind plans (*p* < 0.001) compared to both composite optimization approaches, which were statistically comparable to one another. These findings indicate a superior ability of the TAI-Ind technique to limit intermediate- and low-dose spillage into surrounding healthy brain tissue. However, these dosimetric advantages were accompanied by a trade-off in treatment delivery efficiency, as the increased modulation complexity of the TAI-Ind technique required a significantly greater number of MU (7268.92 ± 1076.94, *p* < 0.001) than the two composite approaches.

### Dosimetric outcomes of OARs

The dosimetric outcomes for OARs across the three planning techniques are presented in **Table 4**. Analysis of the normal brain demonstrated that the absolute volume receiving 12 Gy (V12Gy) was significantly lower with the TAI-Ind technique (35.53 ± 12.48 cm^3^, *p* < 0.001) than with either the GCI-Comp or TAI-Comp techniques, which did not differ significantly from one another. In addition, the median dose (D_50%_) to the normal brain exhibited a statistically significant stepwise reduction across the three techniques, with the lowest value observed in the TAI-Ind plans (322.36 ± 85.77 cGy, *p* < 0.001). For mean brain dose, both TAI-based techniques (TAI-Comp and TAI-Ind) achieved significantly lower values than GCI-Comp (*p* = 0.001), while remaining statistically comparable to each other.

**Table 4:** Dosimetric parameters for organs at risk with each planning technique.

	Mean ± SD (minimum – maximum)			Global P
	GCI-Comp	TAI-Comp	TAI-Ind	
Normal brain^¶^ V12 (cm^3^)	42.27 ± 15.04^a^ (26.52-78.57)	41.02 ± 13.98^a^ (22.80-66.71)	35.53 ± 12.48^b^ (22.26-67.00)	<0.001
Normal Brain^¶^ D_50%_ (cGy)	370.27 ± 98.42^a^ (192.34-593.41)	352.30 ± 98.23^b^ (190.05-556.40)	322.36 ± 85.77^c^ (189.74-502.63)	<0.001
Normal brain^¶^ D_mean_ (cGy)	441.69 ± 87.31^a^ (270.40-605.30)	410.36 ± 103.59^b^ (270.40-629.60)	399.22 ± 86.40^b^ (260.60-559.30)	0.001
Hippocampus D_max_ (cGy)	507.19 ± 183.25^a^ (49.60-660.10)	430.00 ± 132.74^b^ (44.50-609.30)	304.67 ± 82.55^c^ (45.10-400.30)	<0.001
Hippocampus D_100%_ (cGy)	194.28 ± 95.51^a^ (17.25-378.50)	177.15 ± 97.69^a,b^ (17.74-328.60)	158.06 ± 78.44^b^ (16.65-331.00)	0.004
Optic pathway D_max_ (cGy)	409.84 ± 188.86 (50.00-776.40)	403.36 ± 196.31 (90.80-765.60)	345.53 ± 158.71 (126.10-668.50)	0.385
Right Cochlea D_mean_ (cGy)	204.48 ± 104.82^a^ (11.60-339.30)	185.13 ± 93.03^b^ (11.10-317.00)	154.65 ± 90.17^c^ (10.80-327.80)	<0.001
Left Cochlea D_mean_ (cGy)	206.57 ± 113.04^a^ (15.60-350.20)	189.34 ± 106.05^b^ (13.50-334.50)	171.40 ± 98.46^c^ (11.10-321.40)	<0.001
Right Cochlea D_max_ (cGy)	255.29 ± 124.19^a^ (12.40-392.30)	226.29 ± 106.02^b^ (12.00-357.50)	200.00 ± 100.64^c^ (11.60-330.20)	<0.001
Left Cochlea D_max_ (cGy)	258.91 ± 134.99^a^ (16.90-395.90)	231.60 ± 126.02^b^ (16.00-384.30)	208.59 ± 115.70^c^ (13.30-352.30)	<0.001
Right Lens D_max_ (cGy)	234.09 ± 129.92 (16.60-499.20)	211.57 ± 106.73 (25.50-456.30)	210.01 ± 107.86 (34.10-435.00)	0.169
Left Lens D_max_ (cGy)	177.23 ± 89.30 (20.90-456.60)	198.38 ± 111.74 (21.50-493.10)	193.33 ± 109.40 (20.20-494.70)	0.385
Right Orbit D_max_ (cGy)	357.57 ± 189.49 (28.10-789.50)	370.31 ± 150.20 (52.10-687.10)	354.18 ± 155.68 (65.50-706.90)	0.579
Left Orbit D_max_ (cGy)	295.07 ± 116.24 (53.30-550.80)	324.38 ± 120.05 (43.00-581.60)	307.09 ± 115.72 (38.80-564.00)	0.107
Brainstem† D_max_ (cGy)	854.04 ± 327.36^a^ (38.80-1461.90)	735.40 ± 299.52^b^ (46.20-1247.80)	662.11 ± 253.24^b^ (39.10-1222.00)	<0.001
Spinal Canal‡ D_max_ (cGy)	640.15 ± 400.66^a^ (10.50-1361.70)	607.78 ± 345.10^b^ (8.10-1169.60)	507.66 ± 292.41^c^ (7.00-990.00)	<0.001

SD, standard deviation; GCI-Comp, Geometric Center Isocenter with Composite Dose Prescription; TAI-Comp, Target-Anchored Isocenter with Composite Dose Prescription; TAI-Ind, Target-Anchored Isocenter with Individual Dose Prescription; V12 defined as the volume of normal tissue receiving at least an absorbed dose of 12 Gy; cm^3^, cubic centimeter; cGy, centigray; D_max_, minimum dose to the 0.1 cm^3^ volume of the organ receiving the highest doses; a, b, or c, in the comparison between two groups, if the groups have the same letter, there is no statistically significant difference, but if the same letter is not found, there is a statistically significant difference; ^¶^normal brain is whole brain minus gross tumor volume; ^†^the brainstem consists of the midbrain and pons but without medulla oblongata; ^‡^spinal canal also includes the medulla oblongata (17).

For central neural structures, including the hippocampus, brainstem, and spinal canal, the TAI-Ind approach consistently demonstrated the most favorable dosimetric profile. For the hippocampus, D_max_ showed a statistically significant stepwise decrease across the evaluated techniques, reaching its lowest value with TAI-Ind (304.67 ± 82.55 cGy, *p* < 0.001). Furthermore, the D_100%_ for the hippocampus was significantly lower with TAI-Ind than with GCI-Comp, whereas TAI-Comp produced intermediate values that overlapped with both techniques (*p* = 0.004). For the brainstem, maximum dose was significantly reduced with both TAI-Comp and TAI-Ind compared to GCI-Comp (*p* < 0.001), with no significant difference between the two TAI-based approaches. Similarly, maximum dose to the spinal canal demonstrated a statistically significant stepwise reduction, with the lowest exposure observed in the TAI-Ind plans (507.66 ± 292.41 cGy, *p* < 0.001).

A significant dosimetric benefit was also observed for the auditory structures. Both mean and maximum doses to the bilateral cochleae exhibited a statistically significant stepwise reduction across the planning techniques, with TAI-Ind consistently providing the lowest dose exposure (*p* < 0.001). In contrast, no statistically significant differences were observed among the techniques in the maximum doses delivered to anterior structures, including the optic pathways, bilateral lenses, and bilateral orbits (all *p* > 0.05).

**Figure 3:**
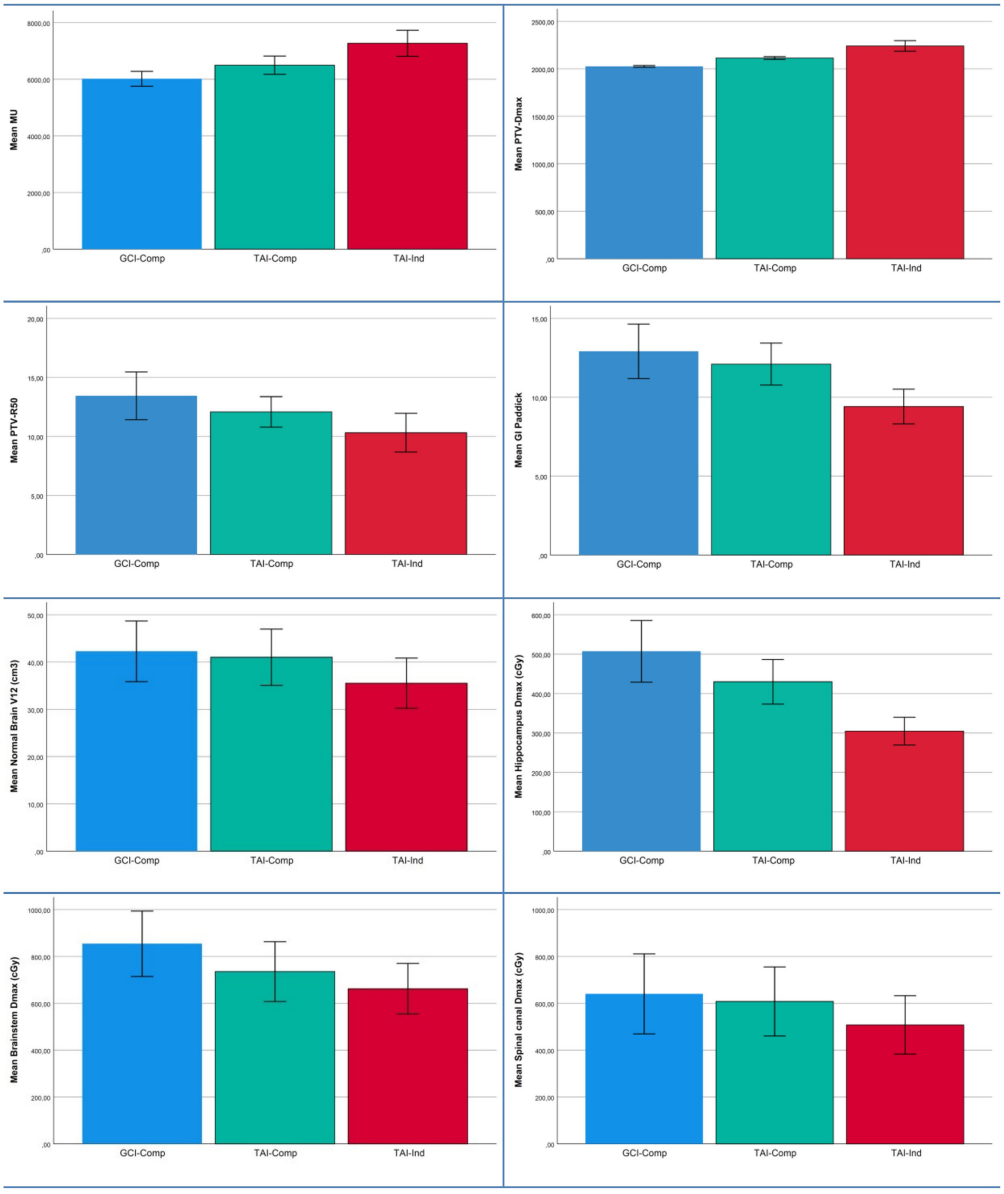
Comparative dosimetric outcomes across GCI-Comp, TAI-Comp, and TAI-Ind for eight key parameters.

Comparative dosimetric outcomes across GCI-Comp, TAI-Comp, and TAI-Ind for eight key parameters are presented in **Figure 3**.

## Discussion

BMs impose a significant neurocognitive burden and demand radiotherapeutic precision with minimal tolerance for planning compromise. SRS has emerged as a cornerstone of multidisciplinary management, offering an effective balance between focal tumor ablation and preservation of neurological function, and its indications have expanded considerably in recent years — from definitive treatment of intact lesions to postoperative, preoperative, and salvage applications (5, 6, 25–27). This growing and diversifying clinical demand has been met largely by modern LINACs, which now play a central role in neuro-oncological practice and account for a rapidly increasing proportion of SRS delivery worldwide (4, 28, 29).

Historically, LINAC-based SRS relied on a sequential multi-isocenter approach, in which each target required a separate isocenter and an independent image-guidance procedure. Although dosimetrically effective, this workflow became increasingly inefficient as the number of metastases increased, significantly prolonging treatment times and patient immobilization. As a result, the treatment of numerous intracranial lesions was often time-consuming and clinically impractical (7, 8). To overcome these limitations, contemporary treatment planning has increasingly shifted toward SIMT-SRS. By utilizing advanced delivery techniques, such as VMAT and dynamic conformal arc therapy, SIMT-SRS enables the simultaneous treatment of multiple spatially separated lesions using a single shared isocenter (3, 9). While positioning this isocenter at the geometric center of all metastases improves treatment efficiency and patient comfort, it also introduces important geometric and dosimetric challenges. In particular, lesions located farther from the isocenter are more susceptible to rotational setup errors, which may compromise peripheral target coverage. In addition, simultaneous MLC modulation across multiple targets can exacerbate the island-blocking effect, resulting in increased low-dose radiation spillage into healthy brain tissue (10). Consequently, achieving a steep dose gradient while minimizing radiation exposure to critical OARs remains one of the principal challenges in SIMT-SRS optimization.

Isocenter placement has, therefore, become a major focus in efforts to optimize SIMT-SRS further. Dosimetric studies have shown that positioning the isocenter at the geometric center is not always optimal (30), particularly when target sizes (31) and spatial distributions (32) vary substantially, as this configuration may necessitate larger margin expansions. To reduce irradiation of healthy tissue and mitigate rotational setup uncertainties, several alternative mathematical strategies for isocenter placement have been proposed. The volume centroid approach weights each target according to its volume, thereby shifting the isocenter toward larger lesions. In contrast, the point centroid method assigns equal importance to all lesions regardless of size, generating a geometrically balanced midpoint among metastases. The inverse volume centroid approach prioritizes smaller lesions and positions the isocenter closer to targets that may be more vulnerable to rotational inaccuracies. Finally, the center-of-surface-area method determines isocenter placement based on lesion surface geometry rather than volumetric characteristics (30, 33). Despite their geometric differences, all of these mathematically derived approaches share an important clinical limitation: the calculated isocenter frequently lies within normal brain tissue and lacks a direct anatomical reference point. Establishing the isocenter within a distinct metastatic lesion, as proposed in our TAI approach, offers a practical and reliable alternative by providing a clear anatomical anchor, enhancing setup robustness, and reducing the likelihood of geometric miss without relying solely on abstract spatial calculations. We specifically selected the metastasis nearest to the geometric center so that the isocenter would remain as close as anatomically possible to the distance-minimizing centroid, while ensuring that the isocenter itself corresponds to tissue requiring irradiation rather than healthy parenchyma. This anatomical constraint, however, necessarily comes at the cost of a modest increase in isocenter-to-target distance compared with the true geometric center, as reflected in our results. Importantly, this modestly greater distance did not translate into inferior target coverage, as PTV-V95% remained statistically comparable across all three techniques, suggesting that the anatomical anchoring benefit outweighed the theoretical risk associated with the modest increase in distance. Nonetheless, this residual distance-related uncertainty should be accounted for in treatment margins and image-guidance verification, consistent with standard SRS practice.

PTV dose prescription methodology represents another critical determinant of plan quality, directly influencing both target conformity and intratumoral dose intensity. According to ICRU Report 91 guidelines, modern radiosurgery prescribes the dose to a specific covering isodose surface, typically the 80% isodose line for LINAC-based platforms, to achieve a higher central tumor dose while maintaining a rapid dose fall-off in surrounding tissues (34). However, conventional SIMT-SRS planning generally applies a single global prescription dose to all targets combined into a unified volume, referred to as the composite PTV. This aggregated approach limits the modulation flexibility required for multiple lesions distributed throughout the brain and often forces planners to balance adequate peripheral target coverage against optimal sparing of normal tissues.

Optimization of treatment parameters on an individualized basis is essential to overcome the dosimetric limitations associated with composite dose prescriptions in conventional SIMT-SRS planning. To address this challenge, we evaluated a novel dual-optimization strategy, TAI-Ind, which combines physical anchoring of the isocenter to the most centrally located metastasis with independent dose prescriptions for each PTV. Our results demonstrate that overall plan conformity and target coverage were maintained across all evaluated techniques, whereas the TAI-Ind approach enabled deliberate intratumoral dose escalation, resulting in statistically significant increases in both maximum and mean target doses. Importantly, this enhanced central dose deposition was not achieved at the expense of damage to normal tissues. Notably, isocenter relocation alone (TAI-Comp) improved several PTV and OAR dose parameters but did not significantly alter R50%, GIPaddick, or normal brain V12Gy, indicating that the steep dose-gradient benefit is primarily attributable to individualized dose optimization rather than isocenter placement alone. Instead, TAI-Ind produced a significantly steeper dose gradient, as evidenced by significantly lower GI_Paddick_ values and a marked reduction in the V12Gy. Consequently, the sharper dose fall-off translated into more favorable dosimetric profiles for critical neural structures, yielding statistically significant reductions in maximum dose exposure to the brainstem, hippocampus, spinal canal, and bilateral cochleae. Although the increased modulation complexity required for individualized target optimization resulted in a significantly higher number of MU, this moderate reduction in delivery efficiency appears to be a reasonable clinical trade-off for gains in both tumor dose intensification and normal tissue protection.

The TAI-Ind approach demonstrated more favorable dosimetric characteristics, particularly regarding dose-gradient sharpness and normal brain sparing, which may offer a dosimetric rationale for future efforts aimed at reducing the risk of radiation necrosis in SIMT-SRS, pending clinical validation. The V12Gy is widely recognized as an important predictor of radionecrosis (35, 36). Although recent comprehensive analyses have recommend limiting V12Gy to 5–10 cm^3^ for single intracranial lesions (36), cumulative V12Gy values inevitably exceed these thresholds when multiple metastases are treated simultaneously (37). For example, Palmiero et al. (10) reported a mean V12Gy of 56.5 cm^3^ using conventional single-isocenter VMAT for multiple targets, which was reduced to 47.3 cm^3^ only through implementation of a more complex and time-consuming semi-regional dual-isocenter technique. In our cohort, the baseline GCI-Comp technique produced a mean V12Gy of 42.3 cm^3^, consistent with previously reported multi-target experiences. Application of the TAI-Ind dual-optimization strategy reduced this volume significantly to 35.5 cm^3^ without requiring additional patient setups or imaging procedures. This reduction in V12Gy was directly associated with the steeper dose gradient achieved by TAI-Ind, reflected by an improvement in the GI_Paddick_ from 12.9 to 9.4. Although a GI of approximately 3 is generally considered optimal for single-target radiosurgery (22), higher values are expected in SIMT-SRS because of the geometric complexity of MLC modulation and unavoidable inter-target dose bridging (10, 38, 39). By anchoring the isocenter within a metastatic lesion and individualizing dose prescriptions, the TAI-Ind technique effectively minimizes inter-target dose spillage, resulting in steeper dose gradients and improved OARs dose sparing comparable to that achieved with substantially more complex or prolonged treatment techniques.

It is crucial to emphasize that the statistically significant dosimetric differences achieved by the TAI-Ind technique directly translate into clinically meaningful benefits. First, the reduction of normal brain V12Gy by approximately 6.8 cm³ (from 42.27 to 35.53 cm³) is substantial. As established in the literature, the individual risk of symptomatic radionecrosis acts as a continuous function of V12Gy; therefore, this absolute reduction effectively mitigates the risk of late neurological toxicities (35, 36). Furthermore, the statistically significant reduction in maximum hippocampal dose observed with TAI-Ind supports the clinical goal of preserving neurocognitive function. As highlighted by the ASTRO Clinical Practice Guidelines for brain metastases, sparing the hippocampal compartment is integral to preventing memory recall deterioration and maintaining quality of life in long-term survivors (11). Lastly, the steeper dose gradients allowed for significant intratumoral dose escalation (higher PTV-Dmax). According to ICRU Report 91 recommendations, achieving a higher central tumor dose via inhomogeneous prescriptions safely enhances the ablative effect and local tumor control without violating surrounding tissue tolerances (34).

Several limitations should be considered when interpreting the findings of this study. First, as a comparative replanning study based on a specific patient cohort from a single institution, the analysis lacks direct clinical outcome data. Although the observed reduction in V12Gy provides a strong dosimetric rationale for a lower risk of radionecrosis, this potential benefit remains theoretical and requires prospective clinical validation to determine its impact on symptomatic radionecrosis, local tumor control, and long-term patient outcomes. Second, achieving the individualized target coverage and steep dose gradients characteristic of the TAI-Ind technique required a higher number of MU. The associated increase in beam-on time may elevate the risk of intrafraction motion, a factor that was not modeled in this static planning study but could potentially affect dose conformity during clinical treatment delivery. Thirdly, all plans were generated by a single planning team; although this design ensured methodological consistency and eliminated planner-dependent variability as a confounding factor, it precludes assessment of inter-planner reproducibility, which should be evaluated in future multi-institutional studies. Finally, all treatment plans were generated using a single treatment planning system and LINAC platform. Given the inherent differences among radiosurgery technologies, the reproducibility and generalizability of the dosimetric advantages observed with TAI-Ind should be validated across other planning systems and treatment platforms.

## Conclusions

The TAI-Ind dual-optimization strategy represents a promising dosimetric refinement in SIMT-SRS. By physically anchoring the isocenter to the metastasis located closest to the geometric center and implementing individualized dose prescriptions for each target, this technique addresses several geometric and dosimetric limitations associated with conventional composite planning. Without compromising target coverage, TAI-Ind provided more favorable dosimetric characteristics, including greater intratumoral dose escalation, steeper dose gradients, and improved sparing of both normal brain tissue and critical neural structures, compared with the composite-prescription approaches. Although the increased MU requirements warrant careful consideration of potential intrafraction motion, the reduction in V12Gy suggests a potential for TAI-Ind to help mitigate radiation-related toxicity. These findings support TAI-Ind as a feasible and clinically promising strategy for the treatment of patients with multiple BMs using SIMT-SRS, pending further validation.

## Author’s note

The English in this document has been checked by at least two professional editors, both native speakers of English. For a certificate, please see: http://www.textcheck.com/certificate/r20ieI.

## Data Availability

The original contributions presented in the study are included in the article/supplementary material. Further inquiries can be directed to the corresponding author.
